# Monomorphic *Trypanozoon*: towards reconciling phylogeny and pathologies

**DOI:** 10.1099/mgen.0.000632

**Published:** 2021-08-16

**Authors:** Guy Oldrieve, Mylène Verney, Kamil S. Jaron, Laurent Hébert, Keith R. Matthews

**Affiliations:** ^1^​ Institute for Immunology and Infection Research, School of Biological Sciences, University of Edinburgh, Edinburgh EH9 3FL, UK; ^2^​ Unité PhEED, Laboratoire de Santé Animale, Site de Normandie, ANSES, RD675, 1443012 Goustranville, France; ^3^​ Institute of Evolutionary Biology, Ashworth Laboratories, School of Biological Sciences, University of Edinburgh, Edinburgh EH9 3JT, UK

**Keywords:** asexual, monomorphism, selection efficacy, *Trypanosoma brucei*

## Abstract

*Trypanosoma brucei evansi* and *T. brucei equiperdum* are animal infective trypanosomes conventionally classified by their clinical disease presentation, mode of transmission, host range, kinetoplast DNA (kDNA) composition and geographical distribution. Unlike other members of the subgenus *Trypanozoon*, they are non-tsetse transmitted and predominantly morphologically uniform (monomorphic) in their mammalian host. Their classification as independent species or subspecies has been long debated and genomic studies have found that isolates within *T. brucei evansi* and *T. brucei equiperdum* have polyphyletic origins. Since current taxonomy does not fully acknowledge these polyphyletic relationships, we re-analysed publicly available genomic data to carefully define each clade of monomorphic trypanosome. This allowed us to identify, and account for, lineage-specific variation. We included a recently published isolate, IVM-t1, which was originally isolated from the genital mucosa of a horse with dourine and typed as *T. equiperdum*. Our analyses corroborate previous studies in identifying at least four distinct monomorphic *T. brucei* clades. We also found clear lineage-specific variation in the selection efficacy and heterozygosity of the monomorphic lineages, supporting their distinct evolutionary histories. The inferred evolutionary position of IVM-t1 suggests its reassignment to the *T. brucei evansi* type B clade, challenging the relationship between the *Trypanozoon* species, the infected host, mode of transmission and the associated pathological phenotype. The analysis of IVM-t1 also provides, to our knowledge, the first evidence of the expansion of *T. brucei evansi* type B, or a fifth monomorphic lineage represented by IVM-t1, outside of Africa, with important possible implications for disease diagnosis.

## Data Summary

The data used in this study are available from the National Center for Biotechnology Information (NCBI) Sequence Read Archive (SRA) and the Wellcome Sanger Institute. The accession numbers can be found in Table S1 (available with the online version of this article).

**Impact Statement**

*Trypanosoma brucei* are unicellular parasites typically transmitted by tsetse flies. Subspecies of *T. brucei* cause human African trypanosomiasis and the animal diseases nagana, surra and dourine. *T.brucei evansi* and *T. brucei equiperdum* have branched from *T. brucei* and, by foregoing tsetse transmission, expanded their geographical range beyond the sub-Saharan tsetse belt. These species can only reproduce asexually and exhibit morphological uniformity in their host (‘monomorphism’). *T. brucei evansi* and *T. brucei equiperdum* have historically been classified based on fragmentary information on the parasites’ transmission routes, geographical distribution, kinetoplast DNA (kDNA) composition and disease phenotypes. Our analysis of genome sequencing data from monomorphic *T. brucei* supports at least four independent origins with distinct evolutionary histories. One isolate, IVM-t1, typed as *T. equiperdum*, is a closer relative to *T. brucei evansi*, highlighting the risk of using pathognomonic descriptors for subspecies assignment. We show clear lineage-specific variation in the selection efficacy in monomorphic *T. brucei*. Using the evolutionary relationships between lineages, we suggest it would be beneficial to reconcile phylogeny and pathology in monomorphic trypanosomes.

## Introduction

The subgenus *Trypanozoon* (*Trypanosoma brucei* spp.) contains parasites of medical, veterinary and economic significance. In their mammalian form, developmentally competent (pleomorphic) trypanosomes transition from a proliferative ‘slender’ form to a cell-cycle arrested ‘stumpy’ form, adapted for survival in the midgut of its vector, the tsetse fly (*Glossina* spp.). Progression to the stumpy form occurs in a density-dependent manner, mediated by a stumpy induction factor [1–4]. Some *Trypanozoon* have a reduced ability to transition from the slender to stumpy morphotype and so are described as ‘monomorphic’. In the field, monomorphic *Trypanozoon* were historically classified as independent species, *T. equiperdum* and *T. evansi*, due to their distinct modes of transmission, geographical distribution, disease phenotype and host range [5]. These monomorphic trypanosomes can infect livestock, and are currently implicated in causing dourine and surra, respectively [6].

More recently, it was proposed that *T. evansi* and *T. equiperdum* are subspecies of *T. brucei* (*T. brucei evansi* and *T. brucei equiperdum*) which had lost part or all of their kinetoplast DNA (kDNA), the parasites’ mitochondrial genome that encodes respiratory components required for viability in the tsetse fly vector [7]. Whole-genome comparisons found that monomorphism arose independently on at least four separate occasions, and further monomorphic isolates could be continuously emerging from pleomorphic *T. brucei* in the field. However, *T. brucei evansi* and *T. brucei equiperdum* are polyphyletic and can be assigned into at least four independently derived lineages, such that their subspecific names do not describe the evolutionary relationships between the different monomorphic *Trypanozoon* [8–10]. *T. brucei evansi* type A and *T. brucei evansi* type B originate from *T. brucei* in Western and Central Africa, whilst *T. brucei equiperdum* type OVI and *T. brucei equiperdum* type BoTat evolved from *T. brucei* in Eastern Africa [9]. Whilst many naming conventions exist, for the remainder of this article we will use the proposition by Cuypers *et al*. (2017) [9], which currently most accurately describes the polyphyletic nature of monomorphic trypanosomes (i.e. *T. brucei evansi* type A, *T. brucei evansi* type B, *T. brucei equiperdum* type OVI and *T. brucei equiperdum* type BoTat).

The four *T. brucei* lineages converged on a monomorphic phenotype accompanied by a switch from cyclical to mechanical transmission. All of the monomorphic subspecies display a reduction or removal of their kDNA alongside an inability to complete their life cycle in their vector, locking these parasites into a tsetse-independent transmission mode [11, 12]. Current evidence suggests that *T. brucei evansi* type A and *T. brucei evansi* type B predominantly rely on transmission via biting flies (e.g. tabanids and *Stomoxys*), whilst *T. brucei equiperdum* type OVI and *T. brucei equiperdum* type BoTat are sexually transmitted between Equidae. Neither are cyclically transmitted via the tsetse vector [6] and, as sexual reproduction occurs in the tsetse salivary gland [13], monomorphic trypanosomes are obligately asexual and proliferate via mitosis [14]. Escape from transmission by the tsetse fly, whose range is restricted to sub-Saharan Africa, has facilitated the expansion of monomorphic trypanosomes to other regions of Africa, Asia, Europe and the Americas, although they have subsequently been eradicated from North America and limited to local outbreaks in Europe [15].

The use of disease pathology, host species, geographical range and kDNA composition can complicate species classification where distinct lineages have converged on a phenotype. Research that treats polyphyletic lineages as a single group may miss more subtle, but important, differences between lineages. Here, we have re-analysed existing genomic data from publicly available monomorphic *Trypanozoon* isolates to confirm their evolutionary relationships. This analysis included a recent isolate from Mongolia, IVM-t1, derived from the genital mucosa of a horse and classified as *T. equiperdum* based on its clinical disease symptoms and host species [16, 17].

Through re-analysing publicly available whole genome data, we found at least four groups of monomorphic *T. brucei* with independent origins, consistent with previously published phylogenies [8, 9]. Our analysis concludes that currently IVM-t1 forms a clade with *T. brucei evansi* type B, despite its clinical presentation being more typical of the conventional description of *T. equiperdum*. The presence of IVM-t1 in the genital mucosa of a horse with signs of dourine supports the hypothesis that there is considerable plasticity in the mode of transmission, host range and clinical presentation of monomorphic *Trypanozoon* strains of distinct origins, as was suggested by Brun *et al.* and Carnes *et al.* [8, 18]. These findings exemplify the need to classify monomorphic trypanosomes based on genetic information, unbiased by the mode of transmission, disease presentation, kDNA composition and host range. We also identified lineage-specific variation in the heterozygosity and efficacy of selection of the four independent monomorphic lineages, highlighting the importance of characterizing phylogeny-informed lineages. Finally, we note that the ancestor of the *T. brucei evansi* type B clade, or a fifth monomorphic lineage represented by IVM-t1, extended its geographical range outside of Africa.

## Methods

### Variant calling

Publicly available genome data were accessed from the National Center for Biotechnology Information (NCBI) Sequence Read Archive (SRA) [19] and the Wellcome Sanger Institute. Samples sequenced with older technologies, such as solid-state ABI, were excluded. When PacBio and Illumina data were available for the same sample, Illumina data were used preferentially to standardize the comparison.

The *T. brucei* EATRO 1125 AnTat 1.1 90 : 13 [20] genome was sequenced as part of this study. DNA was extracted using a DNeasy blood and tissue kit with an RNAse A step (Qiagen), following the manufacturer’s instructions. The DNA was sequenced (HiSeq 4000) and cleaned by BGI (Hong Kong) (7 723 274 reads at 150 bp length). The *T. brucei* EATRO 1125 AnTat 1.1 90 : 13 raw data have been submitted to the NCBI SRA (PRJNA720808). The complete list of genomes analysed in this study, including their accession IDs, are summarized in the Table S1.

The quality of the raw reads was analysed with fastqc (v. 0.11.9) and subjected to quality trimming with trimmomatic (v0.39) [21]. The reads were trimmed with the following filters: SLIDINGWINDOW 4 : 20; ILLUMINACLIP adapters.fa 2 : 40 : 15; MINLEN 25. The trimmed reads were aligned to the *T. brucei* TREU927/4 V5 reference genome [22] with bwa-mem (v. 0.7.17-r1188) [23]. The reads were prepared for variant calling by following the gatk4 (v. 4.1.4.1) best practices pipeline, which included marking duplicate reads [24, 25]. The read recalibration step was performed by initially calling variants on un-calibrated reads with gatk4’s haplotype caller [26]. The top 20 % highest confidence calls from the first round were used as the confident call set to re-calibrate the raw bam files. Variants were then re-called on the recalibrated bam files with gatk4’s haplotype caller [26].

The variants were combined and filtered with the following stringent cut-offs, in keeping with gatk’s best practices pipeline and previous studies [9]. SNPs were filtered by quality by depth (<2.0), quality score (<500.0), depth (<5.0), strand odds ratio (>3.0), Fisher’s exact (>60.0), mapping quality (<40.0), mapping quality rank sum (<−12.5), read position rank sum (<−8.0), window size (10) and cluster size (3). Indels were filtered on their quality by depth (<2.0), quality (<500.0), Fisher’s exact (>200.0) and read position rank sum (<−20.0).

### Phylogenetic analysis

The filtered variants, described above, were filtered again to retain sites where a genotype had been called in every sample, using VCFtools (v. 0.1.16) [27]. These sites were analysed in two ways. The first was based on SNPs that occurred across all of the sites in the *T. brucei* TREU927/4 V5 reference genome and the second from SNPs that occurred in a coding sequence (CDS) of a gene (excluding pseudogenes) found on one of the 11 Mb chromosomes [22]. For both of these analyses, a concatenated alignment of each variant was extracted using VCF-kit (v. 0.1.6) [28]. iq-tree (v. 2.0.3) [29] was used to create a maximum-likelihood tree from homozygous variant sites. Within the iq-tree analysis, a best-fit substitution model was chosen by ModelFinder using models that included ascertainment bias correction (MFP+ASC) [30]. ModelFinder identified TVM+F+ASC+R2 as the best fit for both of the alignments which were subjected to 1000 ultrafast bootstraps generated by UFBoot2 [31]. The consensus trees were visualized and annotated using iTOL (v. 5) [32].

### Genome content

Raw reads were used to predict the heterozygosity, G+C content and genome size of each isolate. This analysis was performed using a *k*-mer counting based approach with Jellyfish (v. 2.3.0) [33] and Genomescope (v. 1). A *k*-mer size of 21 was used [34].

Non-synonymous and synonymous SNP ratios (dN/dS) can be used to estimate the selection pressure upon an organism. The filtered SNPs identified for the phylogenetic analysis were split by isolate using bcftools (v. 1.9) [35], and these were then filtered to remove all non-variant sites using gatk4 SelectVariants (v. 4.1.9.0). The reverse complement of each variant call format (VCF) file was generated using SNPGenie (v. 2019.10.31) [36]. SNPGenie within-pool analysis was then completed on each isolate for SNPs found in the longest CDS site of each gene, excluding pseudogenes, on each of the 11 Mb chromosomes of the *T. brucei* TREU927/4 V5 reference genome [22]. The mean was calculated for each isolate. The full dN/dS results are available in Table S2.

### Molecular markers

Molecular marker sequences were downloaded from the NCBI: SRA (Z37159.2), RoTat1.2 (AF317914), *T*. *evansi* VSG JN2118HU (AJ870487), cytochrome oxidase subunit 1 (CO1) (M94286.1 : 10712–12445) and NADH dehydrogenase subunit 4 (NADH4) (M94286.1 : 12780–14090). bwa-mem (v. 0.7.17-r1188) [23] was used to align raw reads to the molecular markers. A minimum overlap of 50 bp between the read and target sequence was used. The molecular marker presence was confirmed by counting the number of bases in the target sequence covered by reads, representing the breadth of coverage, with SAMtools mpileup [37]. Orphan reads were counted and read pair overlap detection was disabled. The breadth of coverage percentage was visualized with pheatmap (v. 1.0.12) [38]. The ATP synthase γ subunit (Tb927.10.180) was screened for lineage-specific variants identified in the variant calling step. Unless stated otherwise, all figures were plotted using ggplot2 (v 3.3.0) [39] and ggrepel (v. 0.8.1) [40] in R (v. 3.6.1) [41].

## Results

### Monomorphism arose independently at least four times

To determine the evolutionary relationships between monomorphic *Trypanozoon*, genomic data from 17 isolates were aligned to the *T. brucei* TREU 927/4 reference genome. This included publicly available data from monomorphic (*n*=9) and pleomorphic isolates (*n*=8). Across the *T. brucei* TREU 927/4 v5 reference genome, 472 794 variant sites (574 775 unique variant alleles, 370 154 SNPs and 204 621 indels) passed the strict quality filtering steps. These sites were filtered further to identify 244 013 homozygous variant SNPs across the genome. A total of 91 853 of these SNPs were present in a CDS on one of the 11 Mb chromosomes of the *T. brucei* TREU 927/4 v5 reference genome. The SNPs across the whole genome and those in a CDS were used to generate two unrooted phylogenetic trees.

**Fig. 1.**
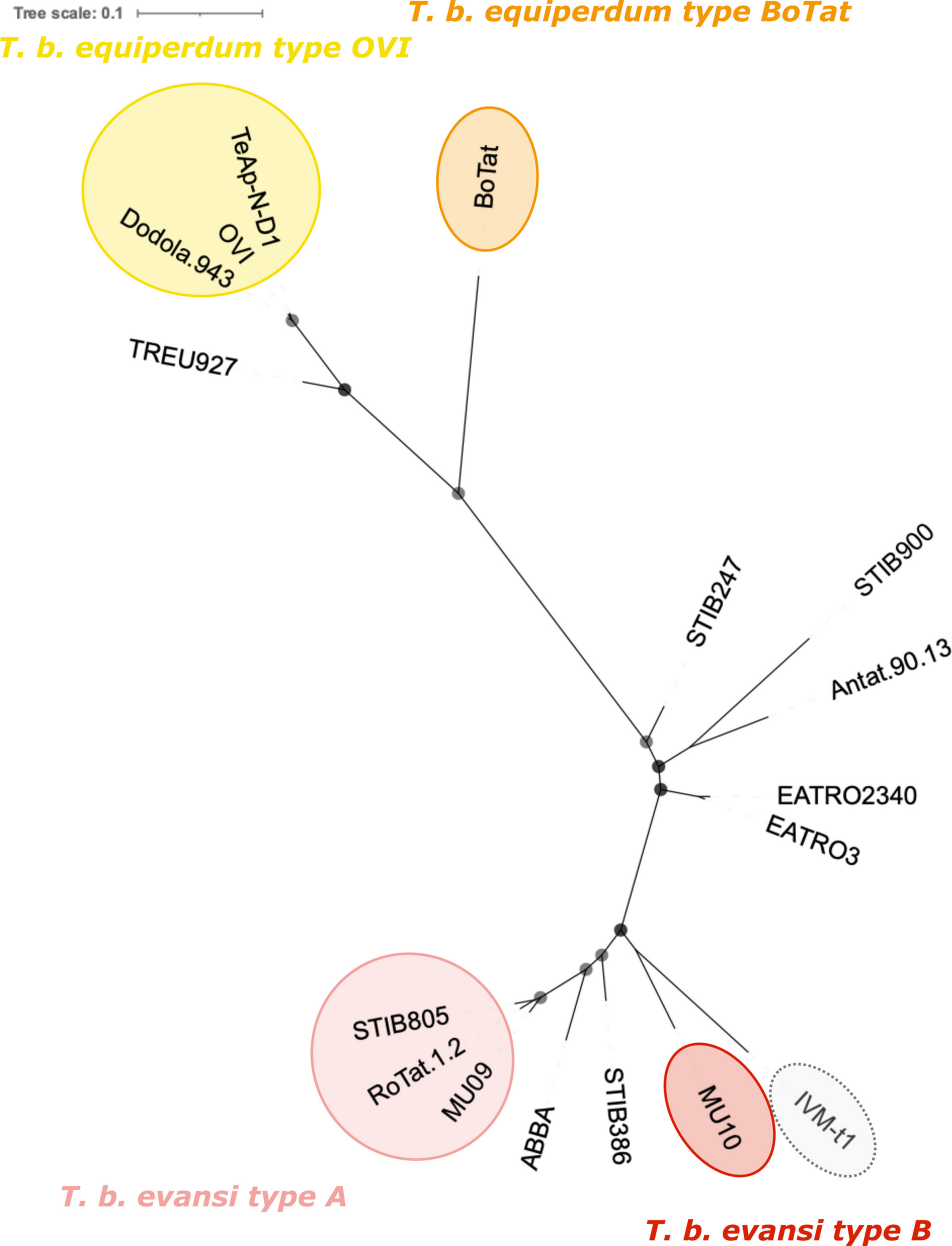
An unrooted phylogenetic tree created with 244 013 homozygous variant SNPs found across the *T. brucei* TREU 927/4 reference genome. The tree was built using a TVM+F+ASC+R2 model. Bootstrap confidence is reported by the size of the grey circles; all bootstrap values were 100 and so each circle is the same size. The branch length scale represents the number of substitutions per site. Monomorphic genomes form four distinct lineages that have expanded from Eastern (*T. brucei equiperdum* type OVI and *T. brucei equiperdum* type BoTat) and Western/Central Africa (*T. brucei evansi* type A and *T. brucei evansi* type B) [9]. IVM-t1 was originally typed as *T. equiperdum*, but groups here with *T. brucei evansi* type B.

Monomorphic *T. brucei* form at least four independent clades (Fig. 1). Our results corroborate previous findings [8, 9] which identified that the *T. brucei equiperdum* type OVI clade arose in Eastern Africa and displays minimal variation between the isolates. *T. brucei equiperdum* type BoTat is separated from other *T. brucei equiperdum* isolates and represents an Eastern African isolate of distinct origin. Trypanosomes designated *T. brucei evansi* also form two discrete clades of Western and Central African origin, with STIB805, RoTat1.2 and MU09 (*T. brucei evansi* type A) displaying low genetic diversity and being distinct from MU10 (*T. brucei evansi* type B).

Interestingly, the Mongolian isolate IVM-t1, with infection and disease characteristics similar to *T. brucei equiperdum*, branched with MU10, which is of West African origin. This contrasts with its previous designation as a *T. equiperdum* isolate, whose ancestors originated from Eastern Africa [9] (Fig. 1). Nonetheless, whilst IVM-t1 and MU10 shared a more recent common ancestor with each other than with any other strain in this analysis, MU10 and IVM-t1 have diverged considerably. Should pleomorphic *T. brucei* isolates be identified that divide the clade composed of MU10 and IVM-t1, the isolates would represent independent clades. The results generated from SNPs found across the whole genome were similar to a tree built from SNPs found in only the CDS, with the former displaying a slight reduction in bootstrap confidence (Fig. S1).

### There is quantifiable variation in the genetic diversity and efficacy of selection between the four asexual monomorphic clades

Asexuality is expected to reduce the heterozygosity of a lineage, although this can be lineage specific [42]. Asexuality can also reduce the efficacy with which selection can act, as reviewed by Otto in 2021 [43]. Efficacy of selection can be estimated by calculating the ratio of nonsynonymous to synonymous variants (dN/dS) compared to a reference genome.

**Fig. 2.**
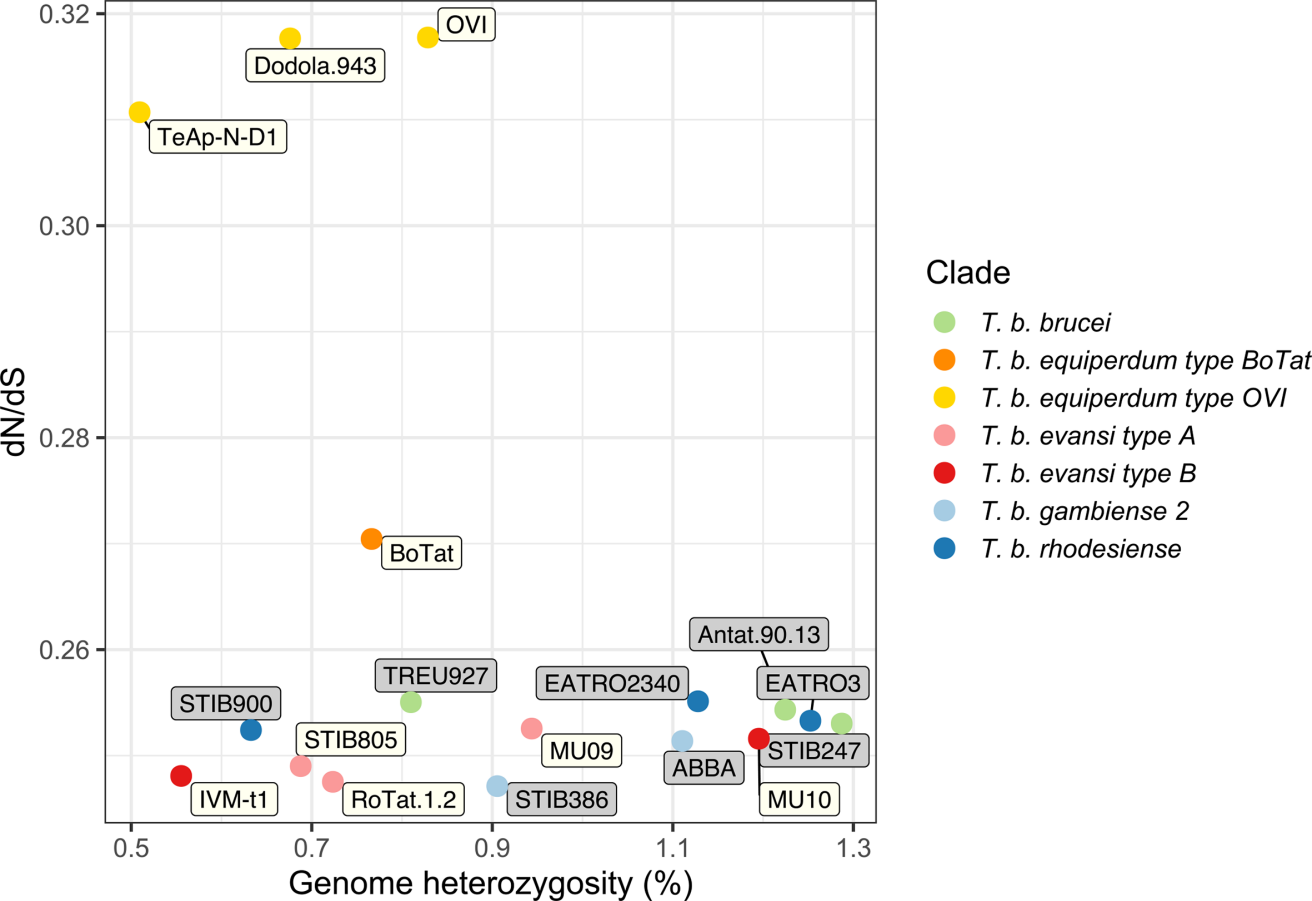
Whole-genome heterozygosity and dN/dS ratio of SNPs present in the longest CDS of every annotated gene, excluding pseudogenes, found on one of the 11 Mb chromosomes of the *T. brucei* TREU 927/4 reference genome. The values were calculated for all publicly available monomorphic isolates and representative pleomorphic isolates. Each point is coloured by clade and the label colour represents a pleomorphic (grey) or monomorphic (white) isolate.

Our analysis demonstrated that monomorphic genomes generally have a lower heterozygosity compared to the genomes derived from pleomorphic isolates (Figs 2 and S2). Notable outliers in this analysis include MU10 (*T. brucei evansi* type B), which has a heterozygosity value closer to that of pleomorphic genomes. The higher dN/dS ratio observed in *T. brucei equiperdum* type OVI is in contrast to the other monomorphic lineages, highlighting the lineage-specific variation, and evolutionary histories, of monomorphic *Trypanozoon*.

Interestingly, IVM-t1 has the second lowest heterozygosity and one of the lowest dN/dS ratios in this analysis, more similar to that of *T. brucei evansi* type A and *T. brucei evansi* type B, and in contrast to the levels observed in *T. brucei equiperdum* type OVI and *T. brucei equiperdum* type BoTat (Fig. 2). Raw reads from *T. brucei brucei* TREU 927/4 were included in the analysis. The variants called against its own genome assembly represent heterozygous loci and misaligned reads. *T. brucei equiperdum* BoTat has the longest branch length in this analysis, but has a far lower dN/dS ratio than the *T. brucei equiperdum* type OVI clade (Fig. S3). The low dN/dS of *T. brucei brucei* TREU 927/4 called against its own genome and the pattern of branch length and dN/dS ratio highlights that the high dN/dS of *T. brucei equiperdum* type OVI is not the result of the evolutionary distance between the clade and the reference genome.

### Genetic markers corroborate phylogenetic and genome content analysis

**Fig. 3.**
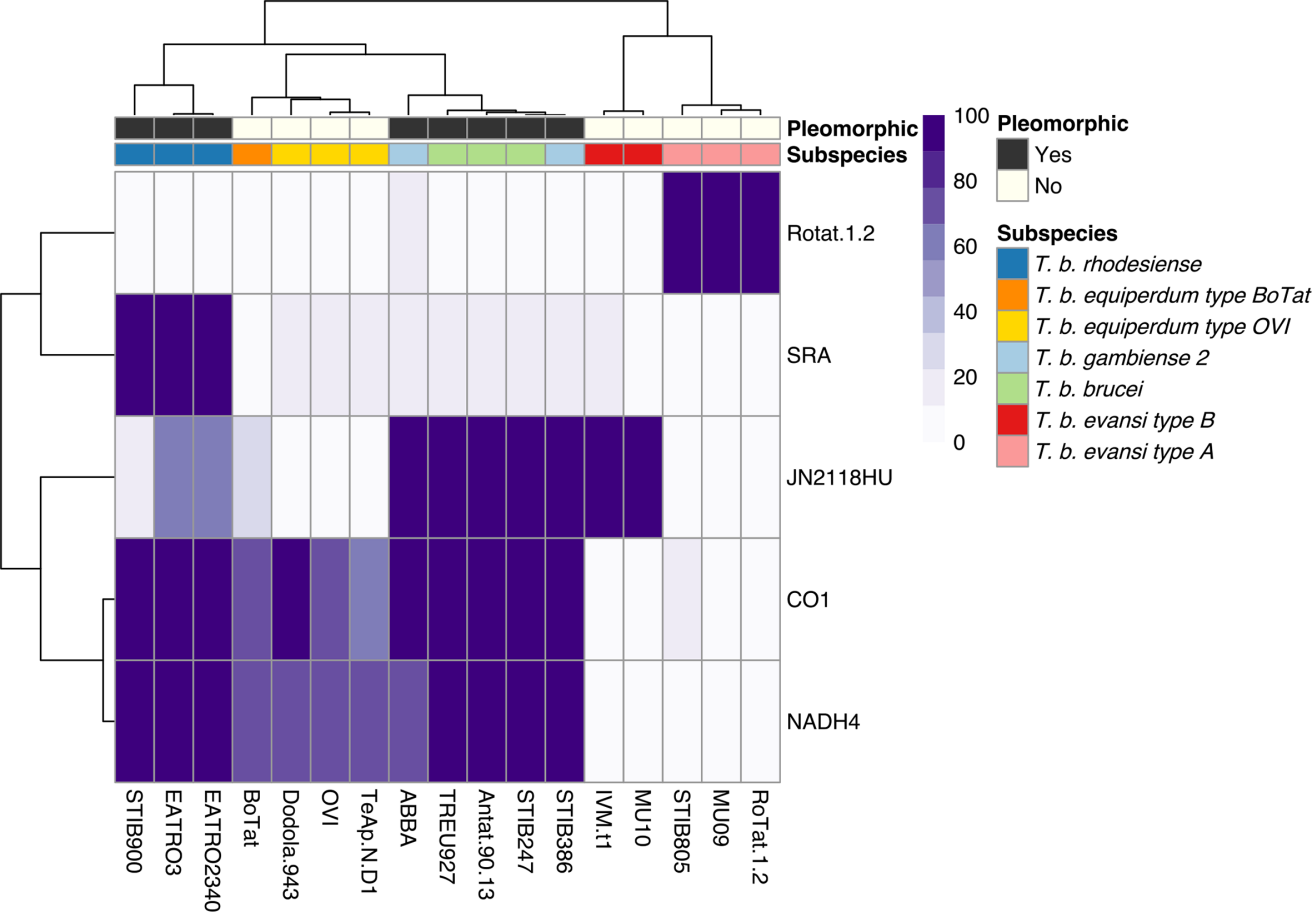
Occurrence of individual genetic markers corroborates the phylogenetic and whole-genome analysis, which highlights at least four independent monomorphic lineages. Genetic markers: SRA (Z37159.2), RoTat1.2 VSG (AF317914.1), JN2118HU VSG (AJ870487.1), cytochrome oxidase subunit 1 (M94286.1: 10712–12445) and NADH4 (M94286.1: 12780–14090). The scale represents the percentage of the marker covered by sequencing reads.

The occurrence of established molecular markers for different *T. brucei* subspecies was compared between the isolates to further validate whole-genome analysis. The markers’ occurrence in each isolate was visualized in a hierarchical clustered heatmap. The clustering highlights a clear distinction between the monomorphic lineages and, furthermore, with just five molecular markers, it is possible to recreate a similar pattern to the phylogenetic tree that is based on whole-genome sequencing data (Figs 1 and 3).

As our phylogenetic and genome content analysis identified a potential discrepancy between the disease description of IVM-t1 and its genotype, particular interest was paid to this isolate. Firstly, being akinetoplastic at the point of sequencing, IVM-t1 lacks coverage of the mitochondrial maxicircle genes cytochrome oxidase subunit 1 (CO1) (M94286.1 : 10712–12445) and NADH dehydrogenase subunit 4 (NADH4) (M94286.1 : 12780-14090), as do *T. brucei evansi* type A and *T. brucei evansi* type B. However, when IVM-t1 was initially isolated, a PCR for NADH4 found that it contained the gene, albeit with a faint signal [16]. Therefore, maxicircle presence in IVM-t1 appears to have been unstable, as is the case in kDNA independent isolates. IVM-t1 reportedly became akinetoplastic after long-term-culture adaptation, as has been observed in other monomorphic isolates [44, 45]. In contrast to IVM-t1, CO1 and NADH4 are present in *T. brucei equiperdum* type OVI and *T. brucei equiperdum* type BoTat genomes.

Secondly, IVM-t1 lacks the RoTat 1.2 VSG (AF317914.1), which is diagnostic for *T. brucei evansi* type A [46], but does have sequence coverage of VSG JN2118HU (AJ870487.1) that is present in *T. brucei evansi* type B, along with some *T. brucei brucei* strains [47, 48]. In contrast, JN2118HU is absent in *T. brucei evansi* type A, *T. brucei equiperdum* type OVI *and T. brucei equiperdum* type BoTat (Fig. 3).

Thirdly, IVM-t1 does not have the M282L ATP synthase γ subunit mutation that has been characterized in other *T. brucei evansi* type B genomes, such as MU10 (Table S3) [48, 49]. This mutation is unable to compensate for kDNA loss [49]. However, IVM-t1 does have two homozygous mutations within that gene that are absent from all other isolates in this analysis (C-terminal genomic codons G817T and A898G), although both are synonymous and so predicted not to influence protein function. In combination, these analyses further support the separation of IVM-t1 from the *T. brucei equiperdum* type OVI and *T. brucei equiperdum* type BoTat lineages, and also highlights differences to *T. brucei evansi* MU10 lineage despite the phylogenetic relationship between the isolates.

## Discussion

*Trypanozoon* phylogeny has historically been based on clinical disease pathology, mode of transmission, geographical range, host species range and kDNA composition, which has complicated the classification of monomorphic trypanosomes and fuelled a long-standing debate in the literature [5]. Here, we re-analyse the molecular phylogeny of monomorphic trypanosomes, providing support for the complete separation of these isolates into at least four clades based on their evolutionary relationships. Our results are consistent with previously published phylogenies of monomorphic *T. brucei* subspecies (Fig. 1) [8, 9]. We consider it is important to classify the monomorphic lineages through their evolutionary relationships because, although they have converged upon a monomorphic phenotype, lineage-specific variation could be missed if their polyphyletic origin is not fully acknowledged. Expanding the repertoire of publicly available genomic data for monomorphic *T. brucei* will negate the limitations of this study.

Variation in heterozygosity can be associated with a transition to asexuality. Asexual taxa can present high heterozygosity if the lineage arose from a hybrid origin, but other types of origin usually lead to a reduction in heterozygosity [42]. Monomorphic strains have lost their tsetse transmission ability. Given that meiotic events occur in tsetse salivary glands, monomorphic strains are obligately asexual and proliferate via mitosis [13, 14]. This asexuality is apparent in the reduction of heterozygosity observed in the majority of monomorphic isolates (Figs 2 and S2). Such reduction in heterozygosity can occur via mitotic recombination and/or gene conversion, with gene conversion having been observed in another asexual *T. brucei* subspecies, *T. brucei gambiense* group 1 [50]. Gene conversion is proposed to reduce or even completely stop the mutational attrition associated with asexuality, and it can occur at a faster rate than the accumulation of spontaneous mutations [51] (Figs 2 and S2).

Notably, *T. brucei equiperdum* type OVI and, to an extent, *T. brucei equiperdum* type BoTat isolates have low heterozygosity and a higher dN/dS ratio than other clades, indicative of a smaller efficacy of purifying selection in removing deleterious alleles. In contrast, the efficacy of selection in *T. brucei evansi* type A and *T. brucei evansi* type B are closer to that observed in pleomorphic lineages. Further, IVM-t1, originally typed as *T. equiperdum*, has one of the lowest dN/dS ratios, more indicative of *T. brucei evansi* type A or *T. brucei evansi* type B. The efficacy of selection can be influenced by the effective population size, which is linked to events during the evolutionary history of a lineage, such as population bottlenecks and variation in the mode of inheritance [52]. Asexuality is predicted to reduce the efficacy with which selection can act [43]. Hence, the observed lineage-specific variation between selection efficacy could be associated with a different length of time as an asexual lineage as the predicted build-up of deleterious mutations is a gradual process, if not completely counteracted by processes like gene conversion. Overall, the analyses of heterozygosity and dN/dS ratio support the different monomorphic lineages displaying contrasting evolutionary histories.

The extremely low heterozygosity of IVM-t1 highlights that when the sample was sequenced, it did not comprise a mixed infection. Furthermore, its low heterozygosity does not support a hybridization-based event at the emergence of the IVM-t1 lineage [42]. Therefore, the long branch and discrepancy in heterozygosity between IVM-t1 and MU10 could be due to an expansion in diversity of the MU10 branch or independent origins of monomorphism within the MU10/IVM-t1 clade. This divergence could have facilitated the distinct transmission mechanism and host range displayed by IVM-t1 with respect to *T. brucei evansi* type B such as MU10. However, at present IVM-t1 and MU10 group as a separate clade and share the monomorphic phenotype. As such, IVM-t1 is currently most accurately described as *T. brucei evansi* type B. As more *T. brucei* are isolated and sequenced, it may be more accurate to define IVM-t1 as a separate clade. In this case, IVM-t1 would represent a fifth independent emergence of monomorphism in *T. brucei*.

The potential expansion of monomorphic lineages, along with the isolation of IVM-t1 from the genital mucosa of a horse with signs of dourine, suggest it could be beneficial to reconcile phylogeny and disease [16]. To fully uncouple the link between phylogeny and disease, studies will be required on the direct mode of transmission of these isolates. For instance, although the presence of IVM-t1 in the genital mucosa of a horse with signs of dourine points towards sexual transmission, it cannot be ruled out that the initial infection was a coinfection of IVM-t1 with an independent *T. brucei equiperdum* type OVI or *T. brucei equiperdum* type BoTat isolate which caused the dourine symptoms but was not recovered after culture. If plasticity in the mode of transmission is established, dourine and surra would best refer to the disease presentation and not the causative agent, particularly where limited clinical information is available for an isolate, precluding an understanding of any variable disease manifestation between individual animals [6].

The emergence of at least four independent monomorphic lineages suggests there could be a selective advantage to monomorphism, at least in the short term. Since monomorphic lineages lose the growth control inherent in the generation of stumpy forms, they display an increase in parasitaemia that improves the chance of non-tsetse transmission when tsetse vectorial capacity is reduced. This adaption to a loss of cyclical transmission could also be the only option for those *Trypanozoon* isolates that become isolated from the tsetse fly through environmental change or geographical relocation of the host [7, 53, 54]. Regardless of the potential adaptive advantage to monomorphism, monomorphic lineages could be constantly emerging from pleomorphic *T. brucei* populations across Africa that remain unidentified due to a lack of sampling. Indeed, as climate change rapidly alters the tsetse flies’ range [55], creating a potential selective advantage for mechanical transmission in areas where tsetse flies are no longer found, the rate of monomorphic *T. brucei* subspecies emergence could increase. Since monomorphic lineages have lost or reduced their growth control mechanism, they can be highly virulent, posing a threat to livestock in their country of origin and with the risk of escape outside traditional disease boundaries.

The analysis of IVM-t1 provides. to our knowledge, the first evidence that *T. brucei evansi* type B, or a fifth monomorphic lineage, has expanded its geographical range outside of Africa. Previously *T. brucei evansi* type B has only been found in Kenya and Ethiopia [47, 48, 56]. The presence of IVM-t1 outside of Africa could complicate the existing screening of animals exhibiting signs of dourine or surra. Currently, the molecular diagnosis of surra and dourine remains limited by the parasitaemia in infected hosts, which can be below the detection limit of parasitological tests and can even be below the detection limit of DNA tests, especially in Equidae and African cattle. Therefore, serological methods are prescribed by the World Organisation for Animal Health for surra and dourine diagnosis. In some regions and for some hosts, for which *T. brucei evansi* type A strains are widely present, the use of serological tests based on the recognition of the specific RoTat 1.2 VSG can provide good sensitivity and specificity [46]. However, this gene is absent in *T. brucei evansi* type B (Fig. 3). Alternatively, surveillance of kDNA integrity remains a useful method of identification, which often aligns with the disease presentation despite these phenomena not being biologically linked. It is important to remember, however, that kDNA integrity comes with the risk of the independent appearance of dyskinetoplasty in multiple lineages or the spontaneous or progressive dyskinetoplasty observed after maintenance *in vitro* culture or in isolates indistinguishable at the level of the nuclear genome.

To avoid these complications, it is possible to rely on markers that are generic for all trypanosome subspecies. Indeed, given that treatment success depends mainly on the stage of the disease rather than the specific *Trypanozoon* lineage [6], we suggest that currently it remains preferable not to aim for a distinction between taxa within the subgenus *Trypanozoon* for first line diagnosis. However, genome sequencing is rapidly reducing in cost whilst improving in portability. Therefore, adaptive genome sequencing represents a promising method for screening of animals infected with monomorphic *Trypanozoon* [57].

We note that although the use of four clades (*T. brucei evansi* type A, *T. brucei evansi* type B, *T. brucei equiperdum* type OVI and *T. brucei equiperdum* type BoTat [9]) attempts to acknowledge the polyphyletic origin of monomorphic *Trypanozoon*, the use of *evansi* or *equiperdum* at the subspecific level is not based on the genetic relationship of the strains. This is the case even when phylotypes are utilized after *evansi* or *equiperdum*. Therefore, we suggest it would be beneficial to break the links between the fragmentary information available for taxonomy, disease phenotype, host range, mode of transmission and the extent of dyskinetoplasty in monomorphic *Trypanozoon*.

Instead, to fully acknowledge their polyphyletic origin and distinct evolutionary histories, we suggest the taxonomy of monomorphic *Trypanozoon* should be based on whole-genome analysis alone, which is quantitative, non-subjective and can be assessed from samples lacking detailed case records. A taxonomy based on the evolutionary relationships between isolates will assist future research by identifying lineage-specific variation in monomorphic *Trypanozoon*. For example, we consider IVM-t1 is currently more accurately classified as a branch of *T. brucei evansi* type B, rather than *T. equiperdum*. Similarly, isolates such as STIB818 (isolated China in 1979) and ATCC 30023 (isolated in France in 1903) were initially classified as *T. equiperdum* but cluster with *T. brucei evansi* [8, 58]. The disease manifestation and tissue specificity of IVM-t1 also suggests that *T. brucei evansi* type B, or a fifth monomorphic lineage, can manifest dourine symptoms with sexual transmission, although direct transmission evidence is needed to confirm this.

The incongruity between the parasites’ evolutionary position and the induced pathology, mode of transmission and tissue tropism highlights the potential for the host physiology and immune response to contribute to clinical disease manifestation, rather than being solely parasite driven [6]. This provides an important exemplar of the potential distinction between the taxonomic position of monomorphic trypanosomes and the diseases they cause.

## Supplementary Data

Supplementary material 1

Supplementary material 2

## References

[1] Vassella E, Reuner B, Yutzy B, Boshart M (1997). Differentiation of African trypanosomes is controlled by a density sensing mechanism which signals cell cycle arrest via the cAMP pathway. J Cell Sci.

[2] Mony BM, MacGregor P, Ivens A, Rojas F, Cowton A (2014). Genome-wide dissection of the quorum sensing signalling pathway in *Trypanosoma brucei*. Nature.

[3] McDonald L, Cayla M, Ivens A, Mony BM, MacGregor P (2018). Non-linear hierarchy of the quorum sensing signalling pathway in bloodstream form African trypanosomes. PLoS Pathog.

[4] Rojas F, Silvester E, Young J, Milne R, Tettey M (2019). Oligopeptide signaling through TbGPR89 drives *Trypanosome quorum* sensing. Cell.

[5] Hoare CA (1972). The Trypanosomes of Mammals: a Zoological Monograph.

[6] Büscher P, Gonzatti MI, Hébert L, Inoue N, Pascucci I (2019). Equine trypanosomosis: enigmas and diagnostic challenges. Parasit Vectors.

[7] Lai DH, Hashimi H, Lun ZR, Ayala FJ, Lukes J (2008). Adaptations of *Trypanosoma brucei* to gradual loss of kinetoplast DNA: *Trypanosoma equiperdum* and *Trypanosoma evansi* are petite mutants of *T. brucei*. Proc Natl Acad Sci U S A.

[8] Carnes J, Anupama A, Balmer O, Jackson A, Lewis M (2015). Genome and phylogenetic analyses of *Trypanosoma evansi* reveal extensive similarity to *T. brucei* and multiple independent origins for dyskinetoplasty. PLoS Negl Trop Dis.

[9] Cuypers B, Van den Broeck F, Van Reet N, Meehan CJ, Cauchard J (2017). Genome-wide SNP analysis reveals distinct origins of *Trypanosoma evansi* and *Trypanosoma equiperdum*. Genome Biol Evol.

[10] Kay C, Williams TA, Gibson W (2020). Mitochondrial DNAs provide insight into trypanosome phylogeny and molecular evolution. BMC Evol Biol.

[11] Schnaufer A, Domingo GJ, Stuart K (2002). Natural and induced dyskinetoplastic trypanosomatids: how to live without mitochondrial DNA. Int J Parasitol.

[12] Dewar CE, MacGregor P, Cooper S, Gould MK, Matthews KR (2018). Mitochondrial DNA is critical for longevity and metabolism of transmission stage *Trypanosoma brucei*. PLoS Pathog.

[13] Peacock L, Ferris V, Sharma R, Sunter J, Bailey M (2011). Identification of the meiotic life cycle stage of *Trypanosoma brucei* in the tsetse fly. Proc Natl Acad Sci USA.

[14] Wheeler RJ, Scheumann N, Wickstead B, Gull K, Vaughan S (2013). Cytokinesis in *Trypanosoma brucei* differs between bloodstream and tsetse trypomastigote forms: implications for microtubule-based morphogenesis and mutant analysis. Mol Microbiol.

[15] Radwanska M, Vereecke N, Deleeuw V, Pinto J, Magez S (2018). Salivarian trypanosomosis: a review of parasites involved, their global distribution and their interaction with the innate and adaptive mammalian host immune system. Front Immunol.

[16] Suganuma K, Narantsatsral S, Battur B, Yamasaki S, Otgonsuren D (2016). Isolation, cultivation and molecular characterization of a new *Trypanosoma equiperdum* strain in Mongolia. Parasit Vectors.

[17] Davaasuren B, Yamagishi J, Mizushima D, Narantsatsral S, Otgonsuren D (2019). Draft genome sequence of *Trypanosoma equiperdum* strain IVM-t1. Microbiol Resour Announc.

[18] Brun R, Hecker H, Lun ZR (1998). *Trypanosoma evansi* and *T. equiperdum*: distribution, biology, treatment and phylogenetic relationship (a review. Vet Parasitol.

[19] Leinonen R, Sugawara H, Shumway M, International Nucleotide Sequence Database Collaboration (2011). The Sequence Read Archive. Nucleic Acids Res.

[20] Engstler M, Boshart M (2004). Cold shock and regulation of surface protein trafficking convey sensitization to inducers of stage differentiation in *Trypanosoma brucei*. Genes Dev.

[21] Bolger AM, Lohse M, Usadel B (2014). Trimmomatic: a flexible trimmer for Illumina sequence data. Bioinformatics.

[22] Berriman M, Ghedin E, Hertz-Fowler C, Blandin G, Renauld H (2005). The genome of the African trypanosome *Trypanosoma brucei*. Science.

[23] Li H (2013). Aligning sequence reads, clone sequences and assembly contigs with BWA-MEM. arXiv.

[24] DePristo MA, Banks E, Poplin R, Garimella KV, Maguire JR (2011). A framework for variation discovery and genotyping using next-generation DNA sequencing data. Nat Genet.

[25] Van der Auwera GA, Carneiro MO, Hartl C, Poplin R, Del Angel G (2013). From FastQ data to high confidence variant calls: the Genome Analysis Toolkit best practices pipeline. Curr Protoc Bioinformatics.

[26] Poplin R, Ruano-Rubio V, DePristo MA, Fennell TJ, Carneiro MO (2017). Scaling accurate genetic variant discovery to tens of thousands of samples. BioRxiv.

[27] Danecek P, Auton A, Abecasis G, Albers CA, Banks E (2011). The variant call format and VCFtools. Bioinformatics.

[28] Cook DE, Andersen EC (2017). VCF-kit: assorted utilities for the variant call format. Bioinformatics.

[29] Nguyen LT, Schmidt HA, von Haeseler A, Minh BQ (2015). IQ-TREE: a fast and effective stochastic algorithm for estimating maximum-likelihood phylogenies. Mol Biol Evol.

[30] Kalyaanamoorthy S, Minh BQ, Wong TKF, von Haeseler A, Jermiin LS (2017). ModelFinder: fast model selection for accurate phylogenetic estimates. Nat Methods.

[31] Hoang DT, Chernomor O, von Haeseler A, Minh BQ, Vinh LS (2018). Ufboot2: improving the ultrafast bootstrap approximation. Mol Biol Evol.

[32] Letunic I, Bork P (2007). Interactive Tree Of Life (iTOL): an online tool for phylogenetic tree display and annotation. Bioinformatics.

[33] Marçais G, Kingsford C (2011). A fast, lock-free approach for efficient parallel counting of occurrences of k-mers. Bioinformatics.

[34] Vurture GW, Sedlazeck FJ, Nattestad M, Underwood CJ, Fang H (2017). GenomeScope: fast reference-free genome profiling from short reads. Bioinformatics.

[35] Narasimhan V, Danecek P, Scally A, Xue Y, Tyler-Smith C (2016). BCFtools/RoH: a hidden Markov model approach for detecting autozygosity from next-generation sequencing data. Bioinformatics.

[36] Nelson CW, Moncla LH, Hughes AL (2015). SNPGenie: estimating evolutionary parameters to detect natural selection using pooled next-generation sequencing data. Bioinformatics.

[37] Li H, Handsaker B, Wysoker A, Fennell T, Ruan J (2009). The Sequence Alignment/Map format and SAMtools. Bioinformatics.

[38] Kolde R

[39] Wickham H

[40] Slowikowski K

[41] R Core Team (2019). R: A language and environment for statistical computing.

[42] Jaron KS, Bast J, Nowell RW, Ranallo-Benavidez TR, Robinson-Rechavi M (2020). Genomic features of parthenogenetic animals. J Hered.

[43] Otto SP (2021). Selective interference and the evolution of sex. J Hered.

[44] Zweygarth E, Kaminsky R, Webster P (1990). *Trypanosoma brucei* evansi: dyskinetoplasia and loss of infectivity after long-term *in vitro* cultivation. Acta Trop.

[45] Kaminsky R, Schmid C, Lun ZR (1997). Susceptibility of dyskinetoplastic *Trypanosoma evansi* and *T. equiperdum* to isometamidium chloride. Parasitol Res.

[46] Claes F, Radwanska M, Urakawa T, Majiwa PA, Goddeeris B (2004). Variable surface glycoprotein RoTat 1.2 PCR as a specific diagnostic tool for the detection of *Trypanosoma evansi* infections. Kinetoplastid Biol Dis.

[47] Ngaira JM, Olembo NK, Njagi ENM, Ngeranwa JJN (2005). The detection of non-RoTat 1.2 *Trypanosoma evansi*. Exp Parasitol.

[48] Birhanu H, Gebrehiwot T, Goddeeris BM, Büscher P, Van Reet N (2016). New *Trypanosoma evansi* type B isolates from Ethiopian dromedary camels. PLoS Negl Trop Dis.

[49] Dean S, Gould MK, Dewar CE, Schnaufer AC (2013). Single point mutations in ATP synthase compensate for mitochondrial genome loss in trypanosomes. Proc Natl Acad Sci USA.

[50] Weir W, Capewell P, Foth B, Clucas C, Pountain A (2016). Population genomics reveals the origin and asexual evolution of human infective trypanosomes. Elife.

[51] Marais GAB, Campos PRA, Gordo I (2010). Can intra-Y gene conversion oppose the degeneration of the human Y chromosome? A simulation study. Genome Biol Evol.

[52] Charlesworth B (2009). Fundamental concepts in genetics: effective population size and patterns of molecular evolution and variation. Nat Rev Genet.

[53] Jensen RE, Simpson L, Englund PT (2008). What happens when *Trypanosoma brucei* leaves Africa. Trends Parasitol.

[54] Schnaufer A (2010). Evolution of dyskinetoplastic trypanosomes: how, and how often?. Trends Parasitol.

[55] Lord JS, Hargrove JW, Torr SJ, Vale GA (2018). Climate change and African trypanosomiasis vector populations in Zimbabwe’s Zambezi Valley: a mathematical modelling study. PLoS Med.

[56] Aregawi WG, Agga GE, Abdi RD, Büscher P (2019). Systematic review and meta-analysis on the global distribution, host range, and prevalence of *Trypanosoma evansi*. Parasit Vectors.

[57] Payne A, Holmes N, Clarke T, Munro R, Debebe B (2020). Nanopore adaptive sequencing for mixed samples, whole exome capture and targeted panels. BioRxiv.

[58] Claes F, Büscher P, Touratier L, Goddeeris BM (2005). *Trypanosoma equiperdum*: master of disguise or historical mistake?. Trends Parasitol.

