# Supplementary material for: Monomorphic Trypanozoon: towards reconciling phylogeny and pathologies
Source: Microb Genom. 2021 Aug 16;7(8):000632. doi: 10.1099/mgen.0.000632 (PMC8549356; doi:10.1099/mgen.0.000632)
Supplement: Supplementary material 1 [file mgen-7-0632-s001.pdf]

Supplementary Material

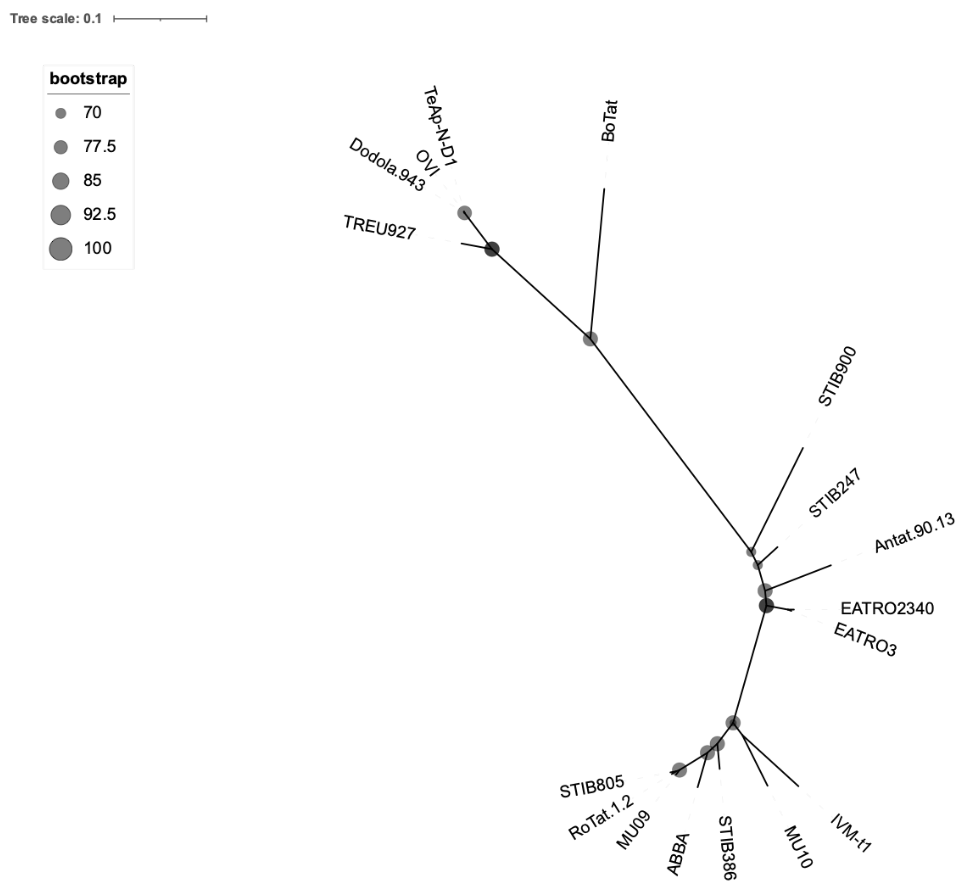

Supplementary figure 1: An unrooted phylogenetic tree created with 91,853 homozygous variant SNPs identified in a CDS on one of the 11 megabase chromosome of the *T. brucei* TREU 927/4 reference genome. The tree was built using the TVM+F+ASC+R2 model. Bootstrap values are reported by circle size. Monomorphic genomes form four distinct lineages which have expanded from Eastern (*T. b. equiperdum* type OVI and *T. b. equiperdum* type BoTat) and Western/Central Africa (*T. b. evansi* type A and *T. b. evansi* type B).

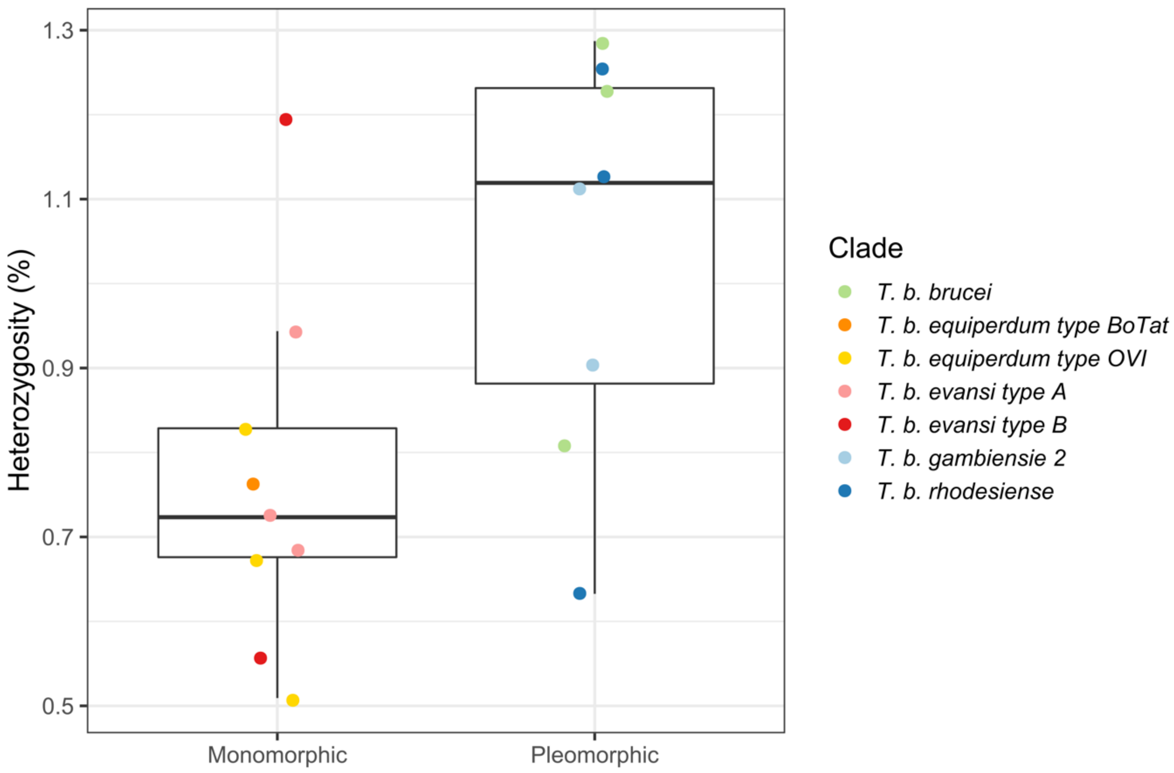

Supplementary figure 2: Whole genome heterozygosity of monomorphic and pleomorphic isolates.

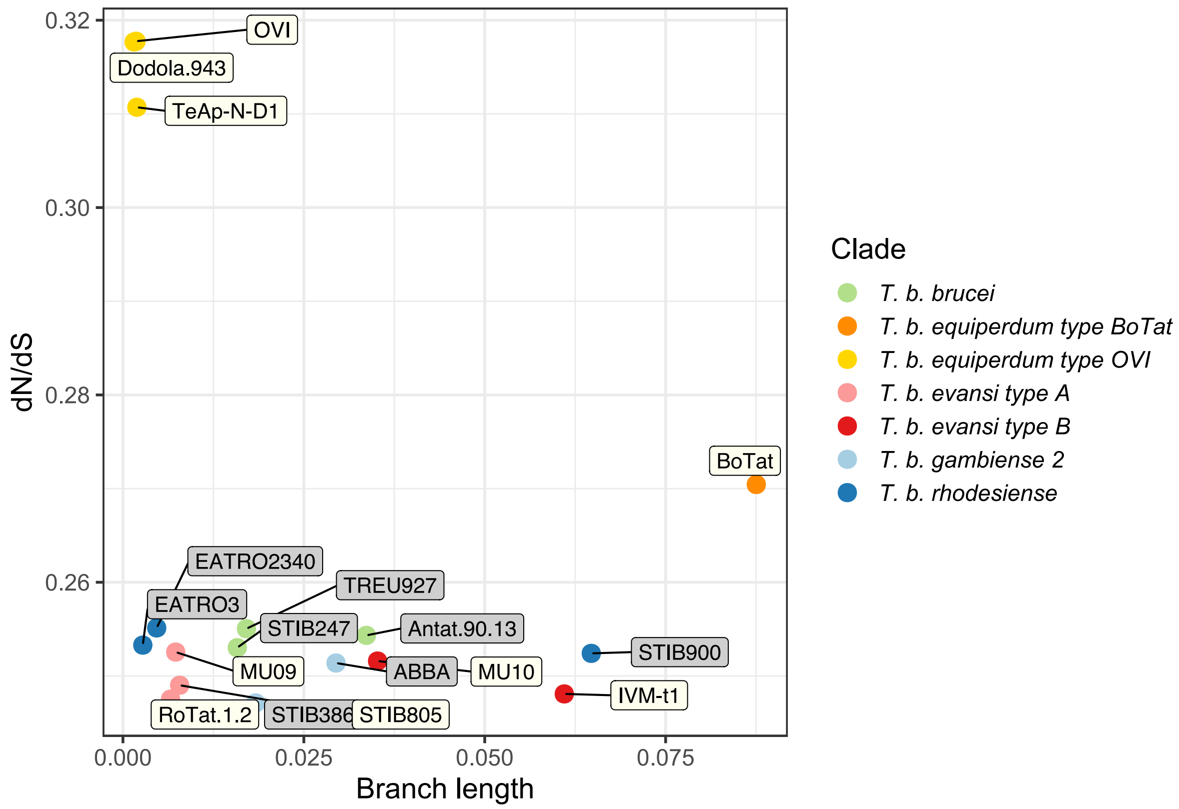

Supplementary figure 3: Branch length and dN/dS ratio of SNPs present in the CDS of genes found on the 11 megabase chromosomes of the *T. brucei* TREU 927/4 reference genome. The values were calculated for all publicly available monomorphic isolates and representative pleomorphic isolates. Each point is coloured by clade and the label colour represents a pleomorphic (grey) or monomorphic (white) isolate.
